# Draft Genome Sequence of *Bacillus amyloliquefaciens* Strain CB, a Biological Control Agent and Plant Growth-Promoting Bacterium Isolated From Cotton (*Gossypium* L.) Rhizosphere in Coimbatore, Tamil Nadu, India

**DOI:** 10.3389/fgene.2021.704165

**Published:** 2021-08-06

**Authors:** Nakkeeran Sevugapperumal, Vimalkumar S. Prajapati, Vanthana Murugavel, Renukadevi Perumal

**Affiliations:** ^1^Department of Plant Pathology, Center for Plant Protection Studies, Tamil Nadu Agricultural University, Coimbatore, India; ^2^Division of Microbiology and Environmental Biotechnology, Aspee Shakilam Biotechnology Institute, Navsari Agricultural University, Surat, India

**Keywords:** *Bacillus amyloliquefaciens*, illumina hi seq, whole genome shotgun sequencing, biocontrol, PGPR, NGS

## Introduction

Although rhizobacteria have been widely explored for their plant growth-promoting capabilities and to manage various fungal and bacterial diseases in plants, viral diseases are an ongoing challenge in the agricultural sector (Vinodkumar et al., 2017). As most plant viral diseases are transmitted through vectors, researchers around the globe are utilizing biotechnological approaches to generate resistant lines. Various antagonistic bio-agents contribute to host defense, and various *Bacillus* species have been shown to produce these agents to protect against a wide range of pathogens. Several reports have demonstrated the antiviral efficacy of various *Bacillus* species against the cotton leaf curl virus (Ramzan et al., 2016), the cucumber mosaic virus in tomato (Zehnder et al., 2000), the tomato mottle virus in tomato (Murphy et al., 2000), and the tobacco mosaic virus in tobacco (Wang et al., 2009).

This bacterium is well known for the production of antibacterial, antiviral, and antifungal substances like *Bacillomyci*n D, *Surfactin*, and *Bacillaene*, which protect the plant from pathogenic organisms (Chen et al., 2009). Additionally, the proteases and amylases produced by *Bacillus amyloliquefaciens* are used in industrial applications (Prajapati et al., 2015, 2017). *Bacillus* species belonging to this group are reported to have 24 diverse antimicrobial peptide (AMP) genes, which lead to the production of numerous compounds such as iturin, bacilysin, bacillomycin, fengycin, surfactin, mersacidin, ericin, subtilin, subtilosin, and mycosubtilin (Chung et al., 2008; Mora et al., 2011). Moreover, *Bacillus* species synthesize various volatile and non-volatile compounds that synergistically restrict plant diseases (Fernando et al., 2005; Mora et al., 2011). *B. amyloliquefaciens* CB has been used to prevent stem rot of carnations, and it was observed that minimum percentage disease incidence and maximum plant growth promotion occurred in plant treated with isolate CB. Further detailed experimentation will be carried out to evaluate the in-depth potential of the *B. amyloliquefaciens* CB.

Here, we report a draft genome sequence of *B. amyloliquefaciens* strain CB, which was isolated from rhizospheric soil of the cotton plant, collected from a cotton farm on the Tamil Nadu Agricultural University (TNAU) campus in Coimbatore, Tamil Nadu, India. This bacterium is gram positive with long rod-shaped, aerobic motile rods arranged singly or in chains. *B. amyloliquefaciens* belongs to the group of free-living soil bacteria, which aid to suppress plant pathogens and assist in promoting plant growth.

## Value of the Data

The *B. amyloliquefaciens* CB draft genome can be used as a base/reference sequence to explore and map specific genes related to AMPs and other important enzymes. It could be a valuable resource to conduct comparative analyses among different species related to *B. amyloliquefaciens*, which may have similar biocontrol properties.

## Methods and Data Analysis

Bacterial DNA from the CB strain was extracted using phenol-chloroform methodology, and purification was performed using a Genomic DNA Clean and Concentrator (Zymo Research, Irvine, CA, USA). One nanogram of highly purified and good-quality DNA was used for the DNA fragment libraries prepared using a Nextera XT DNA sample preparation kit. Sequencing was performed on (2 × 150 paired-end reads with the Illumina v2 reagent kit) (Illumina, San Diego, CA, USA) an Illumina HiSeq system using the standard protocols described by the manufacturer. In total, 4,623,289 reads were obtained, and quality-based read trimming was done using the Trimmomatic software (version 0.30) (Bolger et al., 2014) followed by quality checking with FastQC (version 3.0) (Andrews, 2010). The genome was assembled using GS De Novo Assembler v. 2.6, ABySS v. 1.5.1, Celera Assembler v. 8.3rc2, Edena v. 3.131028, Megahit v. 1.1.2, SOAPdenovo v. 2.04, Velvet v. 1.2.10, SPAdes v. 3.1.1, and SPAdes v. 3.11.0; and the final assembly was merged using CISA v. 1.3 and submitted to the National Center for Biotechnology Information (NCBI) GenBank having accession no. WODE00000000. The total sequence length has been counted up to be 4,113,229 bp consisting of 11 scaffolds/contigs, a contig N50 of 675,513 with L50 of 3, with the largest contig size of the submitted assembly being 1,050,139. The genome size was estimated to be 4.11 MB with a guanine–cytosine (GC) content of 46.30%. Gene annotation was performed using NCBI Prokaryotic Genome Annotation Pipeline (PGAP) (Tatusova et al., 2016), which identified 3,847 protein-coding sequences (Table 1).

**Table 1:** Genomic features of *Bacillus amyloliquefaciens* strain CB annotated using National Center for Biotechnology Information—Prokaryotic Genome Annotation Pipeline (NCBI-PGAP) v. 4.10.

**Items**	**Counts**
Total genes	4,012
Total CDS	3,911
Coding genes	3,847
Coding CDS	3,847
Genes (RNA)	101
rRNAs	8, 8, 7 (5S, 16S, 23S)
tRNAs	73
ncRNAs	5
Total pseudo genes	64

**Figure 1:**
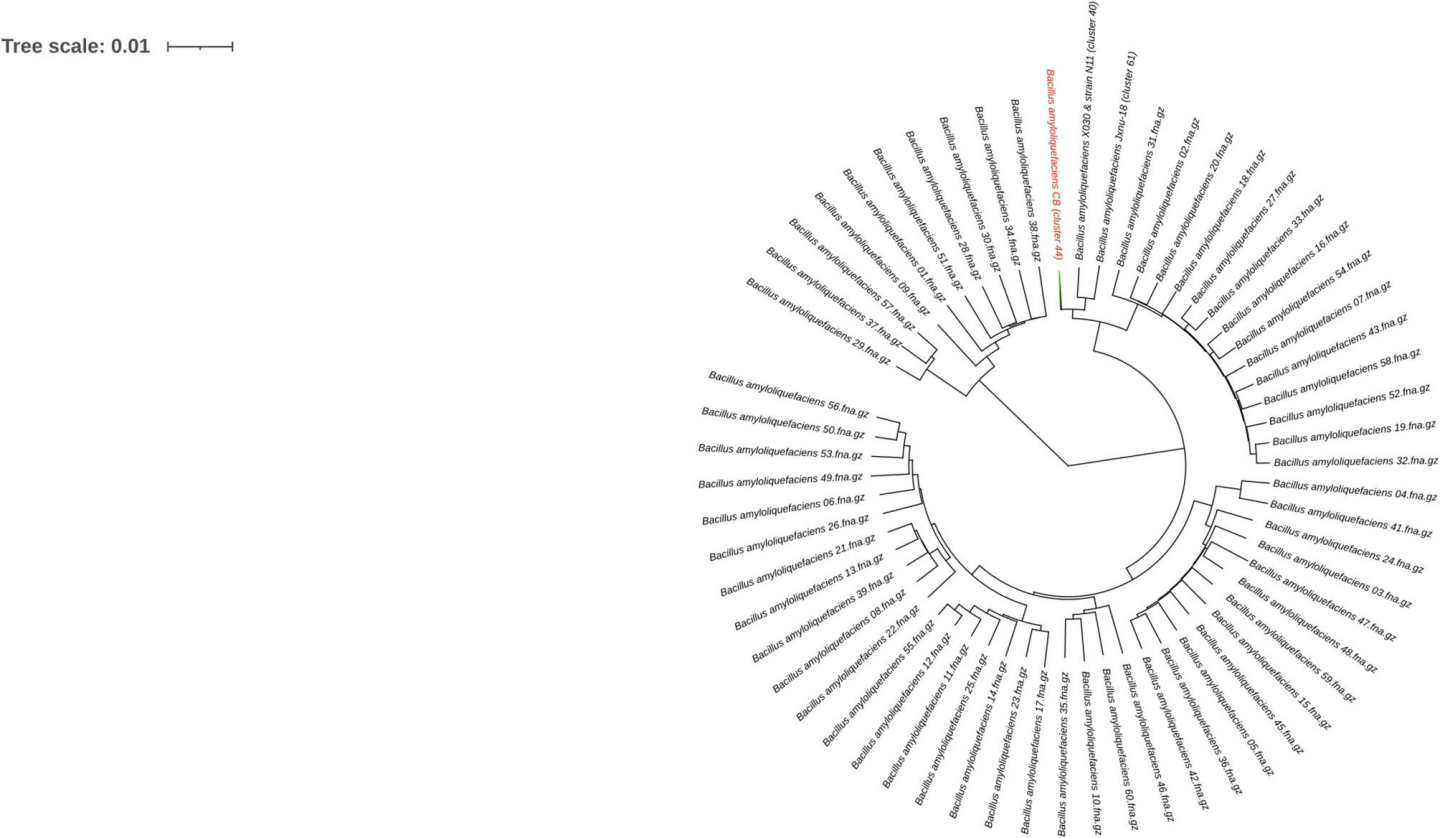
Genome lineage tree of *Bacillus amyloliquefaciens* strain CB. BIONJ algorithm was used to construct the Newick format tree from the distance matrix of all the clustered assemblies (119 different strains of *B. amyloliquefaciens*). The generated Newick tree file was analyzed using the Interactive Tree Of Life (iTOL) v. 5 (strain CB highlighted in red color text with light green color branch).

To infer the phylogenetic relationship, all the 119 assemblies (Supplementary File 1) of *B. amyloliquefaciens* accessible in the NCBI database were considered for the Bacsort analysis (https://github.com/rrwick/Bacsort) including the *B. amyloliquefaciens* CB. A total of 61 clusters of the considered assemblies were generated (cluster accession provided in Supplementary File 2), and FastANI was employed to generate the matrix of all pairwise distance between the clusters. FastANI algorithm (https://github.com/ParBLiSS/FastANI) generates pairwise Average Nucleotide Identity (ANI) measurements using the only sequence shared by two assemblies (Supplementary File 3), which makes it less swayed due to the accessory genome and produce more accurate trees. The phylogeny tree was created by BIONJ algorithm with bootstrap value of 1,000 to form the generated data and was drawn precisely using Interactive Tree Of Life (iTOL) v5, which is an online tool for the display, annotation, and management of phylogenetic trees (Letunic and Bork, 2021) (Figure 1). Out of 61 clusters, two distinct nodes were generated, in which 51 leaves and 10 leaves form a separate group. Fifty-one leaves split into another group having 17 leaves and 34 leaves, which generate other groups consequently as shown in Figure 1. The *B. amyloliquefaciens* strain CB forms a separate cluster (44) having branch length 0.00582, while its nearby cluster (40) includes two strains, *B. amyloliquefaciens* X030 and *B. amyloliquefaciens N11* (branch length 0.00456), while the cluster (61) comprises the *B. amyloliquefaciens* strain Jxnu-18 (branch length 0.00520). Clusters 44, 40 and 61 originated from a common node having branch length 0.00141 (Figure 1).

**Figure 2:**
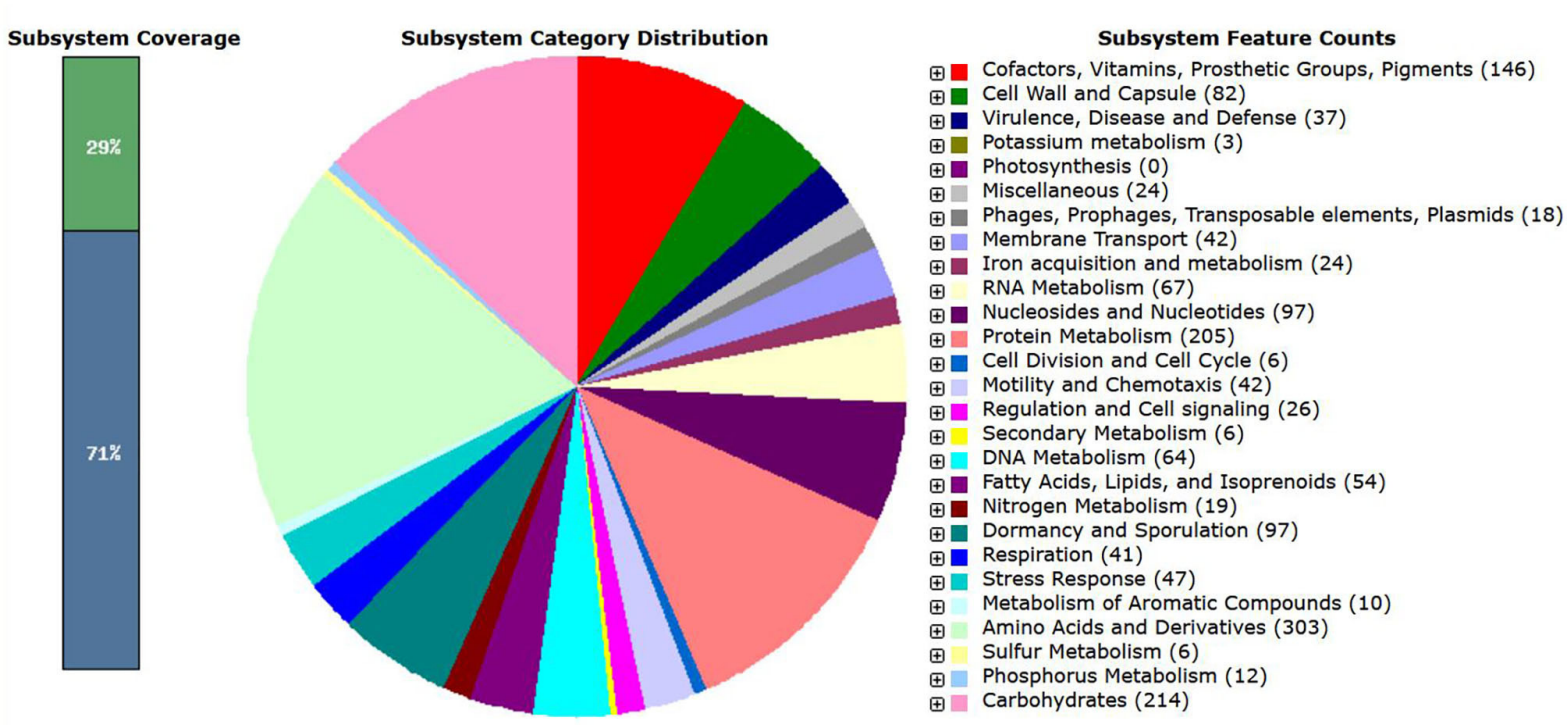
Schematic overview of subsystem coverage, distribution of subsystem category, and subsystem feature counts anticipated in *Bacillus amyloliquefaciens* strain CB using RAST server.

The genome of *B. amyloliquefaciens* CB was also mapped to the seed subsystem to obtain the high-quality genome annotation through Rapid Annotation using the Subsystem Technology (RAST; version 2.0) (http://rast.nmpdr.org) (Overbeek et al., 2014). The total 325 subsystem with 29% subsystem coverage resulted for *B. amyloliquefaciens* strain CB through RAST server (Figure 2). The present investigation revealed that highest number of the genes was allocated to the subsystem category of amino acids and derivatives (303 genes) followed by carbohydrates (214 genes); protein metabolism (205 genes); cofactors, vitamins, prosthetic groups, and pigments (146 genes); nucleosides and nucleotides (97 genes); dormancy and sporulation (97 genes); cell wall and capsule (82 genes); RNA metabolism (67 genes); DNA metabolism (64 genes); fatty acids, lipids, and isoprenoids (54 genes); stress response (47 genes); motility and chemotaxis (42 genes); membrane transport (42 genes); respiration (41 genes); and virulence, disease, and defense (37 genes). A total of 24 genes were found to be associated with iron acquisition and metabolism as well 24 genes for some other miscellaneous applications. More precisely in the category of miscellaneous application, 10 genes were specifically associated to iron–sulfur cluster assembly, five genes for niacin-choline transport and metabolism, and one gene for single-rhodanese-domain proteins. A total of 26 genes were found to be associated with regulation and cell signaling, while 18 genes were collectively specified for phages, prophages, transposable elements, plasmids, and 12 genes for phosphorus metabolism.

Most of the *Bacillus* spp. belonging to this group of genera have been reported to have antifungal potential and have been utilized for the management of the various fungal diseases; however, their efficacy against viral diseases is still not known (Vinodkumar et al., 2018). The PGAP annotation confirms that the *B. amyloliquefaciens* strain CB genome has gene locus srfAA, srfAD, srfAB, and srfAC, which produce various peptides like surfactin non-ribosomal peptide synthetase and surfactin biosynthesis thioesterase. It has been well documented that lipopeptides like surfactin have acquired more attention due to their high surface activity and antibiotic potential. Moreover, surfactin also possesses antiviral, antitumor, and hemolytic activities (Wang et al., 2010), which required further intensive experimentation for characterization to understand its exact mechanism for such action.

The whole-genome shotgun sequence of *B. amyloliquefaciens* strain CB and its annotation report presented here provide a resource for comparative analysis with other genera of *Bacillus* and can be used for engineering purposes where characteristics of the strain CB are desired. The genome representation of *B. amyloliquefaciens* strain CB showed antagonistic potential due to various AMPs imparting various properties like antifungal, antibacterial, and antiviral as well plant growth promotion, leading to strong future prospects for uplifting the sustainable agriculture.

## Data Availability Statement

Bacillus mayloliquefaciens strain CB, whole genome shotgun sequencing project data have been deposited at DDBJ/ENA/GenBank under the accession number WODE00000000. The version described in this data report is the first version having accession number WODE00000000.1. The assembled contigs and its annotation files (CDS, gff, and proteins) are available in https://www.ncbi.nlm.nih.gov/assembly/GCA_011754125.1#/st repository with all the annotations details in Readme file.

## Author Contributions

NS: funding and modeling the study. VP: genome assembly, annotations, and analysis. VP and NS: manuscript preparation. VM and RP: sampling and sequencing and other miscellaneous stuff. All authors contributed to the article and approved the submitted version.

## Conflict of Interest

The authors declare that the research was conducted in the absence of any commercial or financial relationships that could be construed as a potential conflict of interest.

## Publisher's Note

All claims expressed in this article are solely those of the authors and do not necessarily represent those of their affiliated organizations, or those of the publisher, the editors and the reviewers. Any product that may be evaluated in this article, or claim that may be made by its manufacturer, is not guaranteed or endorsed by the publisher.

## Supplementary Material

Supplementary File 1 List of the all the genomes/assemblies of *B. amyloliquefacines* considered in the presented study (strain, biosample and bioproject infromation, assembly level, GC%, Scafold, CDS).

Supplementary File 2 Cluster assemblies: Cluster of assemblies were generated with removing the redundancy of assemblies. Similar assemblies are forming one cluster and have only a single representative chosen based on assembly N50 (Cluster_accessions file lists the cluster name, followed by a tab, followed by a comma-delimited list of the assemblies in that cluster, with the representative assembly marked with a ^*^).

Supplementary File 3 Distance matrix: FastANI produces pairwise ANI measurements of all the generated assemblies's cluster using only the sequence shared by two assemblies.
